# The speed and phase of locomotion dictate saccade probability and simultaneous low-frequency power spectra

**DOI:** 10.3758/s13414-024-02932-4

**Published:** 2024-07-24

**Authors:** Lydia Barnes, Matthew J. Davidson, David Alais

**Affiliations:** School of Psychology, The University of Sydney, Sydney, NSW Australia

**Keywords:** Locomotion, Methods: VEP, EEG, Saccades, Perception and action

## Abstract

Every day we make thousands of saccades and take thousands of steps as we explore our environment. Despite their common co-occurrence in a typical active state, we know little about the coordination between eye movements, walking behaviour and related changes in cortical activity. Technical limitations have been a major impediment, which we overcome here by leveraging the advantages of an immersive wireless virtual reality (VR) environment with three-dimensional (3D) position tracking, together with simultaneous recording of eye movements and mobile electroencephalography (EEG). Using this approach with participants engaged in unencumbered walking along a clear, level path, we find that the likelihood of eye movements at both slow and natural walking speeds entrains to the rhythm of footfall, peaking after the heel-strike of each step. Compared to previous research, this entrainment was captured in a task that did not require visually guided stepping – suggesting a persistent interaction between locomotor and visuomotor functions. Simultaneous EEG recordings reveal a concomitant modulation entrained to heel-strike, with increases and decreases in oscillatory power for a broad range of frequencies. The peak of these effects occurred in the theta and alpha range for slow and natural walking speeds, respectively. Together, our data show that the phase of the step-cycle influences other behaviours such as eye movements, and produces related modulations of simultaneous EEG following the same rhythmic pattern. These results reveal gait as an important factor to be considered when interpreting saccadic and time–frequency EEG data in active observers, and demonstrate that saccadic entrainment to gait may persist throughout everyday activities.

## Introduction

Perception in real-world contexts often takes place in the service of actions executed to achieve behavioural goals. Actions such as visual search, reaching and locomotion all require perception and attention so that they can be effectively executed. Our most frequent action is to move our eyes, and we make upwards of 150,000 saccades per day (Carpenter, 1988, 2000), but we also take several thousand steps each day as we move about our environments. We are therefore inherently active beings, which necessitates the study of perception and related behavioural and neural effects during activity. Despite this, most of what we know comes from studies in which participants are immobile and required to suppress head and eye movements. A number of recent studies, however, have begun to study vision and related behaviours in more active contexts (Cao & Händel, 2019; Cao et al., 2020; Chen et al., 2022; Gramann et al., 2014, 2021; Gwin et al., 2011).

One impetus behind the push to study vision in active contexts comes from relatively recent work showing that locomotion boosts cortical responsivity in the rodent primary visual cortex (Kaneko et al., 2017; Vinck et al., 2015). These effects of locomotion are robust and widely reported (Erisken et al., 2014; Fu et al., 2014; Lee et al., 2014; Mineault et al., 2016; Niell & Stryker, 2010; for a review, see Parker et al., 2020), and studies have begun to appear in recent years exploring these effects in human subjects. Several studies have used peddling on a stationary exercise bicycle to study the role of activity on perception and behaviour. Despite the clear-cut results in rodent studies, these studies have produced mixed results. Bullock et al. (2015) found faster reaction times in an odd-ball detection task (but no change in detection performance) and a larger and earlier P1 component in low-intensity (but not high-intensity) exercise relative to stationary. Bullock et al. (2017) inferred orientation-selectivity from scalp recordings using an inverted encoding model, finding narrower bandwidths for low-intensity cycling relative to stationary, with a corresponding improvement in orientation discrimination. In a visual search study, Dodwell et al. (2021) found faster reaction times during low- and high-intensity peddling and evidence from evoked response potentials (ERPs) of reduced distractor interference.

Other studies of vision in active contexts have focused on walking. In one, Benjamin et al. (2018) measured both electroencephalography (EEG) and visual contrast psychophysics but found no evidence for increased spontaneous firing rate or contrast sensitivity when walking on a treadmill. In a study of free-walking participants on the other hand, Cao and Händel (2019) measured EEG and visual performance and found that walking boosted peripheral visual processing. The evidence supporting this was a relative reduction in the steady-state visually evoked potential (SSVEP) amplitude evoked from a central target during walking compared to standing, and behavioural data showing greater surround-suppression on central target detection during walking. A very recent study by Davidson et al., (2023, 2024) introduced a new fine-grained approach in which free-walking subjects responded to visual stimuli while their gait was tracked so that performance could be calculated at different points within the gait cycle. Results showed a cyclic modulation of visual sensitivity during walking which saw performance rise and fall at a frequency matched to the step rate (or twice the step rate in some participants). In all participants, the sensitivity cycle was phase-locked to the gait cycle, with peaks in the swing phase and troughs at footfall. The discovery that perception modulates up and down within each step might explain the mixed results reviewed above because previous walking and peddling studies averaged performance over extended periods without tracking the action phase, which would cancel the modulation.

These studies underscore how perception changes as we walk. Here, we begin to probe the mechanism by which walking alters our visual responses by observing eye movements during the step cycle. Considering in tandem these routes through which we seek visual input, and their neural bases, is a critical step towards understanding how we see the world. In particular, we seek to understand whether there is a phasic link between gait, saccades and EEG power spectra. Saccade onset is often timed to coordinate with other actions, such as when reaching or pointing (Neggers & Bekkering, 2002) or during an eye blink (Evinger et al., 1994). Earlier work has shown that during visually guided stepping, eye movements generated prior to each step may be used to guide footfall (Hollands & Marple-Horvat, 2001; Matthis & Fajen, 2014). Interestingly, this coupling persists in the dark, with a consistent time delay between saccade onset and step initiation – even when the saccades cannot gather information relevant to step placement (Cao et al., 2020; Hollands & Marple-Horvat, 2001).This suggests a persistent interaction between visuo-motor and locomotor actions that may impact on information-seeking behaviour, and persist during everyday tasks performed simultaneously with walking. Two limitations of this earlier work are that saccades were always task-relevant to guide stepping (Hollands & Marple-Horvat, 2001), or step counts themselves were relatively low (~ 1 min of walking; Cao et al., 2020), precluding an analysis of a generalisable link between saccade onsets and locomotor behaviour in fine detail. Here, leveraging a recently developed continuous probing and position tracking method (Davidson et al., 2024), we hypothesised that saccade onset would be entrained to locomotor rhythm, and investigated whether this entrainment persists at both natural and slow walking speeds.

We designed a task that did not require saccades to aid visually guided stepping and presented visual stimuli at a continuous rate in a head-fixed display, to isolate whether the previously identified interaction between saccade onsets and locomotor phase would persist during the completion of an independent visual task. More specifically, given that saccades coordinate with stepping actions to guide foot placement, and that this coordination persists in the dark (Hollands & Marple-Horvat, 2001), we predict that saccades will entrain to the rhythm of footfall despite a constant target presentation rate and constant viewing distance that precludes eye movements to changing world locations. By using a head-fixed target display, we thus avoid the additional confound of changes in eye movements that occur when approaching a stationary (world-fixed) target and test whether visuomotor and locomotor movements are entrained when no specific eye movements are required to guide locomotion. In line with previous research (Cao et al., 2020; Hollands & Marple-Horvat, 2001), we predict saccades will be more likely around the time of stance phase, rather than in the swing phase in approach to footfall. We test for this while recording simultaneous EEG to document changes in time–frequency power when time-locked to step-cycle phase (and saccade onset) across a broad range of frequencies, to extend previous work investigating changes in alpha-power at a single walking speed (Cao et al., 2020). While this previous research has quantified changes in alpha band power around the time of heel-strike, the dynamics in other frequency bands and comparison of time–frequency EEG data between walking speeds is relatively unexplored. Theta band activity has been implicated in the maintenance of balance and posture (Stokkermans et al., 2022a, 2022b; Stokkermans et al., 2022a, 2022b) and both the theta (3–7 Hz) and alpha (8–12 Hz) ranges have been implicated in perceptual and attentional processes (Helfrich, 2018; Helfrich et al., 2018). As the strength of frontocentral theta may reflect top-down control of attention (Helfrich, 2018), and alpha power can bias visual perception (Samaha et al., 2020), we hypothesised that these frequency bands would be modulated at both walking speeds by step-cycle phase, due to the increasing evidence of cyclic changes to perception within the stride-cycle (Davidson et al., 2024).

To preview the results, we found clear modulations of the likelihood of saccade onset within the gait cycle with a higher incidence around the time of footfall (despite a constant target presentation rate, and no need for visually guided stepping). During both slow and natural walking speeds, the time–frequency transform of EEG data showed periods of increased and decreased power linked to gait phase that were mostly evident in the theta and alpha bands. These findings demonstrate how walking can alter visual perception by altering the moment at which we initiate an eye movement. Together, they provide a proof-of-concept for uniting time-resolved measures (EEG, motion tracking, eye tracking) to better understand how our brains support naturalistic behaviour, and motivate research into the cognitive and motor underpinnings of gait-saccade synchrony.

## Methods

### Participants

We recruited 37 healthy volunteers (14 male, age 21.81 ± 5.29 years). Participants were fluent English speakers and had normal or corrected-to-normal vision. The final sample consisted of 19 participants (six male, age 21.05 ± 3.43 years). This study was approved by the University of Sydney Human Research Ethics Committee (HREC 2021/048). All participants provided informed consent prior to participation and received either course credit or 20 AUD per hour of their time.

### Apparatus and virtual environment

The virtual environment was built in Unity (version 2020.3.14f1) incorporating the SteamVR Plugin (ver 2.7.3; SDK 1.14.15), on a DELL XPS 8950, with a 12th Gen Intel Core i7-12700 K 3.60 GHz processor, running Microsoft Windows 11. The virtual environment consisted of a light green sky and green ground plane. Two overlaid walkways stretched across the ground plane. A longer walkway, in light red, extended 7 m. Superimposed on the centre of this walkway sat a shorter walkway, in dark red, extending half that distance.

Participants walked along this simulated 7-m (or 3.5-m) track wearing the HTC Vive Pro Eye with integrated head-mounted display (HMD) and a wireless adapter kit (130 g weight). Two wireless hand-held controllers were carried to collect participant responses using a right index finger trigger button. The HMD houses two 1,440 × 1,600 pixel (3.5-in. diagonal) AMOLED screens, with a 110° field of view refreshed at 90 Hz. We recorded gaze origin and gaze direction information using the integrated Tobii eye-tracking technology and SRanipal SDK (v1.1.0.1). Five HTC Base Stations (v 2.0) were used to record the 3D coordinates of the HMD, gaze-origin and gaze-direction at 90-Hz resolution. Participants also wore an HMD-compatible mobile EEG system (DSI-24, Wearable Sensing), which wirelessly transmitted data to Wearable Sensing’s bespoke acquisition software running on a Microsoft Surface tablet. Triggers to mark task events in the EEG data were transmitted from the stimulus computer to a NeuroSpec MMBT-S trigger box, which took serial input via a USB cable and output triggers via a D-SUB 25 parallel port cable. The signal was routed through Wearable Sensing’s trigger box before being wirelessly transmitted to the EEG headset. Data and trigger information were saved to the tablet for offline processing.

### Procedure

Participants were first invited to read the information sheet and ask questions before giving informed consent. Next, participants were asked to sit while a tester fitted a mobile EEG system to their head. The system (DSI-24, Wearable Sensing) consists of a rigid frame with adjustable straps around and across the head. The tester centred the system on the participant’s head and tightened it for stability. Participants were asked to identify any pressure points, and the tester adjusted tightness accordingly. The tester then rotated the dry electrodes so that they pushed through the participant’s hair. The electrodes consisted of a ring of small teeth on a rigid arm, with a small spring between the electrode and the rigid arm for flexible fit. The tester adjusted the electrodes until the teeth sat cleanly against the scalp, with an impedance below 10 kΩ. Two supplementary flat electrodes were attached to the earlobes with clips. Participants were invited to view their EEG and observe the effect of face movements on the data.

Once the EEG system was in place, the tester fitted the HMD over the top. The HMD attached to the EEG system with velcro straps, which were tightened for stability and comfort. Some participants’ head shape required that the frontal strip of EEG electrodes be moved up to allow the HMD to fit snugly against their face. The tester then strapped the wireless adapter of the HMD to the top of the participant’s head, and gave them a hand-held controller.

Participants were shown the virtual environment and given time to familiarise themselves with it, while the tester ran an eye-tracking calibration. Participants were informed that they could safely move along the red walkways in the virtual space, and that the experimenter would always be present to ensure they were far from obstacles in the testing room.

Participants began with five static practice trials. They were told what would happen in a trial and given time to ask questions. Each practice trial began with a warning, the text “Ready?” presented centrally in front of the participant for 300 ms. This was followed by a fixation cross presented alone for 500 ms, and a 5-s rapid serial visual presentation (RSVP) stream. The stream consisted of 25 images, 24 of them unique within the trial, one of them (the target) a repetition of the image before. Each image was displayed for 200 ms (100 ms presentation, with a 100-ms gap). Images could appear at any one of eight locations arranged in two quadrants, close to or far from fixation, and repeats were shown at a random location and image number in all trials (drawn from a uniform distribution). The fixation cross remained in place throughout the stream. Participants were instructed to pull the trigger button under their index finger as quickly as possible when they saw a repetition, that is, the target image. At the end of the stream, they had 3.5 s to rest before the next “Ready?” warning. The tester guided the participant through the practice trials and confirmed that they understood the basic trial structure before moving on. The tester then explained the main task and started the main testing session.

The main session consisted of three blocks of 100 trials each. Each block corresponded to one condition: static, slow walking, or natural walking. The static condition was identical to the practice trials described above. In the two walking conditions, the stimulus content and timing were the same as in the static condition. Here, however, participants were asked to walk a prescribed distance during the RSVP stream. In the natural walking condition, participants walked the length of the 7-m light red walkway while viewing the 5-s RSVP stream. In the slow walking condition, participants walked the length of the 3.5-m dark red walkway. Viewing the 5-s RSVP stream while walking a 7-m distance resulted in a walking speed of approximately 1.4 m/s, a preferred speed identified in prior research when walking in flat environments over long distances (Hausdorff et al., 1996) and in natural environments (Hausdorff et al., 1996; Matthis et al., 2018). Completing the same 5-s task over the much shorter 3.5-m distance was chosen to produce the distinctly slow walking speed of 0.7 m/s. Participants were invited to sit, rest, and loosen the HMD in between blocks to minimise eye strain.

### Stimuli

Stimuli were taken from the THINGS image database (Hebart et al., 2019). We selected 30 concepts with high concreteness and frequency ratings: arm, baby, ball, bean, bed, bird, camera, cup, dog, ear, finger, fish, flower, gun, hair, horse, leaf, lion, missile, paper, pig, rifle, sand, snake, spider, syringe, tiger, toilet, tree, worm. For each concept, we selected ten exemplars creating a stimulus set of 300 images. Each image was presented eight times within a condition (24 unique images per trial × 100 trials), excluding presentations as the target image.

**Fig. 1:**
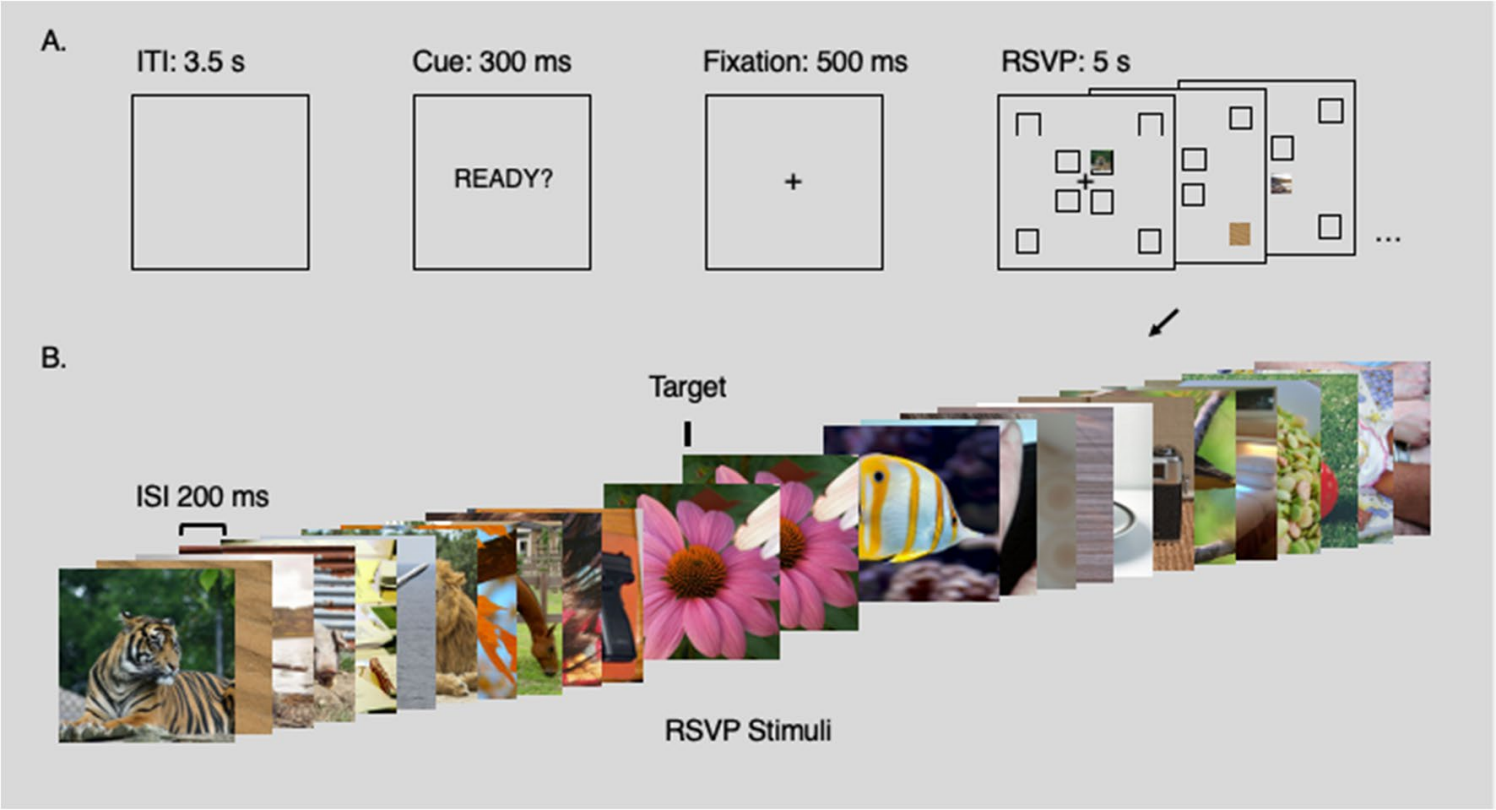
Paradigm. **Panel A** shows the core components of the trial. Trials were separated by a substantial gap to allow participants time to adjust their position between walking lengths of the room. A cue warned participants to settle into their start position. This was followed by a brief fixation cross before the rapid serial visual presentation (RSVP) stream. Stimuli could appear in any one of eight positions, as marked on the figure (the square location markers for illustration only). **Panel B** shows an example set of stimuli for a trial. Each trial included 25 images, 24 of them unique, one of them a target, presented at 5 Hz. Each image was presented for 100 ms, with a blank gap of 100 ms between images. Participants were instructed to press a button whenever they saw an image presented twice in succession (the second presentation is referred to as the target)

Stimuli were presented at a distance of 1 m in front of the participant in the virtual space, in one of eight locations. Four of these locations were close to the centrally presented fixations, with the centre of the image 4 degrees of visual angle away from the centre of the fixation cross, diagonally up-left, up-right, down-right, or down-left. The remaining four locations were similarly placed on the diagonal, but peripherally, with the centre of the image 12 degrees of visual angle from the centre of the fixation cross. Stimuli were 12.25 cm tall and wide, subtending 7 degrees of visual angle (see Fig. 1).

### Eye-movement data

Eye movements were calibrated at the start of each experiment using the manufacturer’s 4-point calibration procedure. Additional calibration was performed after any change to the headset (such as after breaks, between blocks). Continuous recordings of gaze origin and gaze direction were coordinated in 3D coordinates (each frame of all trials at 90-Hz resolution), the following preprocessing steps were used to identify microsaccades following the algorithm of (Engbert & Kliegl, 2003).

First, whole-trial time-series of the eye-position and eye-origin data were tested for outliers. Blinks were identified on the basis of outliers when the difference in position data between successive frames exceeded ± 0.8 m. Raw time-series data were then linearly interpolated from -100 ms to + 100 ms after each blink. Identical windows were interpolated for the gaze origin and gaze direction time-series on the x, y and z axes. After blink interpolation, we applied a two-dimensional (2D) velocity-based algorithm to identify saccades within each trial. Time series of eye-position data were transformed to velocities and smoothed with a five-sample (55 ms) moving average to suppress noise. Microsaccades were then detected as outliers exceeding velocity thresholds that were computed separately for the x and y time series. Following Engbert and Kliegl (2003), we multiplied the median estimator by six times the standard deviation of each velocity time series to protect each threshold computation from noise, and set a minimum duration for each threshold crossing of 22 ms (two samples of our velocity time-series). As each threshold in this algorithm is computed relative to the standard deviation on each trial, it is robust to different noise levels between trials, conditions and participants (Engbert & Kliegl, 2003). We retained the onset, duration and raw position of each microsaccade that was detected for allocation into our stride-based epochs, as detailed below.

### Gait extraction from head-position data

We applied a peak-detection algorithm to the time-series of vertical head position data to estimate the phases of the stride-cycle. As the vertical centre of mass follows near-sinusoidal changes during walking, troughs correspond to when both feet are placed on the ground during the double support stance phase (Gard et al., 2004; Hirasaki et al., 1999; S. T. Moore et al., 2001; Pozzo et al., 1990). We epoched all individual steps based on these troughs, and normalised step-lengths for stride-cycle analysis by resampling the time-series data to 200 data points (0–100% stride-cycle completion, increment of 0.5%). After peak detection, each trial was also visually inspected to identify trials for exclusion, based on wireless signal drop-out or poor gait extraction.

### Cycles-per-stride analyses

We assigned target- and saccade-onsets to their position in the simultaneously occurring stride cycle (i.e., a percentile from 1–100% stride-completion). We then averaged target onsets and saccade counts within 40 linearly spaced bins (with zero overlap) and applied Fourier fits to detect the presence of oscillations.

Significant oscillations were detected via a two-step procedure. We first fitted a sequence of Fourier series models within a forced frequency range to the observed group-level data. These models stepped from 0.2 to 10 cycles per stride, in 0.2 increments, using MATLAB’s curve fitting toolbox and the equation:

$$f(t) = a0 + a1\times cos(wt) + b1 \times sin(wt) = Acos(wt+ \phi )$$

where *a0* is a constant, *a1* and *b1* are cosine and sine coefficients, *t* is time and *w* is the periodicity per stride-cycle. *A* and $\phi$ are the resulting amplitude and phase for the sinusoidal fit. For each forced fit at each frequency, this routine implemented a non-linear least-squares method (400 iterations), from which we retained the goodness of fit (*R*^2^) as our critical value. As a second stage, we assessed the likelihood of these fits occurring by chance by comparing them to a null distribution obtained from a non-parametric shuffling procedure. For each participant the data at a given percentile was selected from another percentile at random (without replacement), and the group-level fitting procedure was repeated as described above. The *R*^2^ values from 1,000 permutations of this procedure were used to quantify the upper 95% confidence interval (CI) of the null distribution at each frequency, and the presence of a significant oscillation was inferred when the observed *R*^2^ value exceeded this upper bound.

### Participant-level analysis and Bayesian population prevalence

At the participant level we performed the same fitting procedure to test for saccadic entrainment between 0.2 and 10 cycles per stride. This enabled a test of Bayesian inference of population prevalence, which uses the results of participant level null hypothesis significance tests (NHSTs) as a complement to the population average effect size (Ince et al., 2021). This method estimates the within-participant replication probability and quantifies the maximum a posteriori (MAP) estimate from this posterior distribution, returning the population prevalence. The estimated population prevalence resides within a CI of uncertainty, and here, following the recommendations of Ince et al. (2022), we report the MAP and the 95% highest posterior density intervals (HPDIs), within which the MAP resides with 95% probability.

Using the participant-level fits we also calculated the phase distribution of saccadic entrainment across participants at slow and natural walking speeds. We tested the distributions for phase clustering using Rayleigh’s test of non-uniformity (Berens, 2009).

### EEG recording and pre-processing

EEG was recorded with a dry headset (DSI-24, Wearable Sensing) from 19 standard electrode positions corresponding to the international 10–20 system (Fp1, Fp2, F7, F3, Fz, F4, F8, T7, C3, Cz, C4, T8, P7, P3, Pz, P4, P8, O1, O2), and two linked earlobe electrodes (A1, A2) for offline re-referencing. Electrode impedances were kept below 20 kΩ during recording, sampled at 300 Hz and referenced online to Pz. During pre-processing, EEGs were re-referenced to the average of A1 and A2, and bandpass-filtered between 0.1 and 40 Hz prior to epoching per trial. Bad channels were removed with pyprep deviation and correlation metrics. Each trial was epoched -100 ms to 6,500 ms relative to trial onset, and baseline-corrected using the 100-ms pre-trial period. Across participants, a total of 11 trials were dropped during epoching (maximum of two per participant) due to a data drop-out in the epoch.

### Gait-cycle-based EEG

For gait-cycle-based EEG analyses, each step was epoched from -600 ms to + 600 ms relative to step cycle onset and completion, respectively. Outliers were identified for exclusion when the epoch standard deviation exceeded 1.5 interquartile ranges above or below the upper and lower quartiles of the distribution of epoch standard deviations across all trials. This method removed an average of 11.5 (*SD* = 17.5) and 37.2 epochs (*SD* = 34.8) in the slow and natural walking speeds across subjects. To investigate time–frequency activity relative to position in the gait-cycle, we convolved our time-series signal with a set of complex Morlet wavelets. Each Morlet wavelet was defined by a complex sine wave tapered by a Gaussian and ranged from 3 to 40 Hz in 50 linearly spaced steps. As wavelet peak frequency increased, the full-width at half-maximum ranged from 600 to 100 ms (Cohen, 2019). After conversion to the time–frequency domain, single-trial gait epochs were resampled from 1–100% cycle completion to allow comparisons, with an additional ± 30% buffer (260 data points, in increments of 0.5%). Due to the cyclic nature of locomotion, time–frequency power within each frequency was normalised to percent change over the entire epoch using the full epoch as a baseline.

### Saccade-locked EEG

We epoched saccade-locked EEG activity from -600 to + 1,100 ms relative to saccade onset. Saccade epochs that began before RSVP onset or exceeded the trial duration were omitted from analysis. We applied the same outlier-detection and Morlet-wavelet convolution as described above, with no additional resampling in units of time. To facilitate comparisons, saccades were additionally sorted based on their occurrence within a co-occurring step cycle into four quartiles (1–25%, 25–50%, 51–75%, 76–100% step cycle completion). For brevity these quartiles were combined into the approximate stance and swing phases to realign saccade-locked EEG relative to the dominant features of step-cycle EEG modulations. All saccade-locked time–frequency power was normalised to percent change over the entire epoch, using a baseline computed from the average of all saccades (irrespective of gait quartile).

For all EEG analyses we corrected for multiple comparisons using non-parametric cluster-based permutation tests (Maris & Oostenveld, 2007). For time–frequency data, contiguous *t*-scores from dependent-samples *t*-tests were retained which exceeded *p* < 0.01 (uncorrected) and compared to null distributions obtained using Monte Carlo permutation tests (2,000 repetitions). Each permutation exchanged data labels at random, and the maximum sum of contiguous *t*-scores was retained to create the null distribution of clustered test statistics (Maris & Oostenveld, 2007). By comparing the observed cluster statistic to the empirical cumulative of the null distribution we finally obtained the cluster-level p-value (reported here as *p*_cluster_) corrected for multiple-comparisons.

### Exclusions

Thirty-seven participants took part in the study. One participant found the set-up uncomfortable, and finished before the task was completed. Two participants’ behavioural data were not properly recorded. Three were excluded for substantial EEG drop-out, and seven for EEG trigger failure. Finally, five were excluded for missing eye-tracking data, leaving 19 participants with full EEG, eye-tracking and behavioural data. All results reported below are from these 19 intact datasets.

## Results

We designed an engaging rapid serial visual presentation (RSVP) task for participants to view while we simultaneously recorded eye-movement data and mobile-EEG during slow and natural walking speeds (Fig. 1). We included an oddball task in which participants were warned that there could be a target in each trial, indicated by the immediate repetition of an image in the RSVP stream (see *Methods*). Although approximately 50% of targets were missed (likely due to our instructions, which did not imply that there would be a target in every trial), false alarm rates were low (that is, only 10.2% of responses preceded the target). Critically, response times were numerically similar between conditions (mean and 95% CI: static = 755.8 ± 94.5 ms; slow walking = 742.5 ± 75.8 ms; natural walking = 811.4 ± 81.8 ms), and were not statistically different (static vs. slow walking: *t*(18) = 0.18, *p* = 0.86; static vs. natural walking: t(18) = -1.06, *p* = 0.30; slow walking vs. natural walking: *t*(18) = -1.01, *p* = 0.33). Having established that participants were engaged with our RSVP task, we next turn to our main focus regarding the characteristics of the simultaneous eye-movement data and EEG, and their interaction with the phases of the stride-cycle.

### Walking speed alters gait and gaze dynamics

**Fig. 2:**
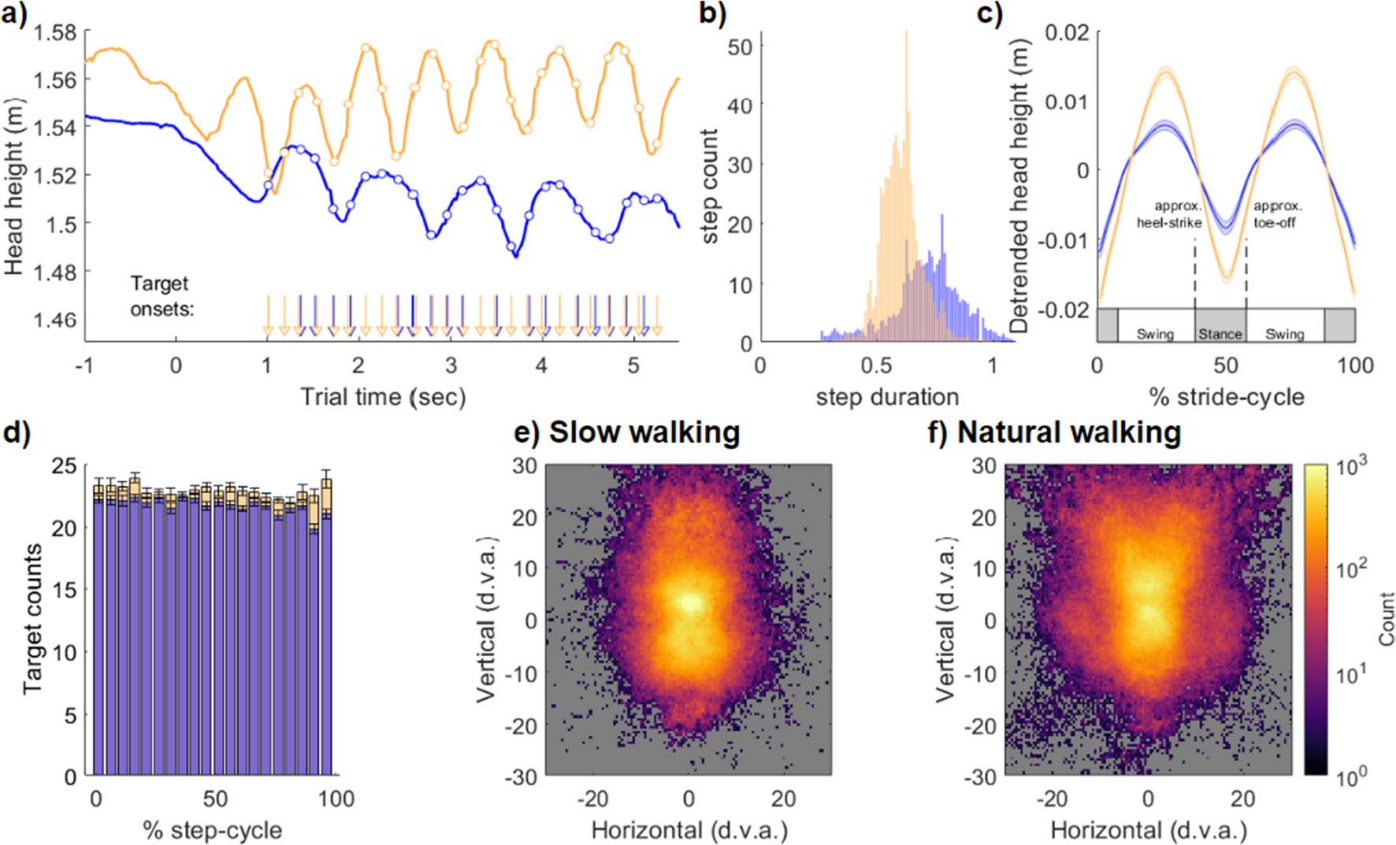
Gait extraction and gaze direction at slow and natural walking speeds. **(a)** Example trials from one participant at the slow (blue) and natural (yellow) walking speeds. Raw head height is displayed from which the peaks and troughs are used to define each step. The sinusoidal head position for the normal (yellow) and slow (blue) walking speeds displays the characteristic increase in step duration (cf. panel b) and decrease in height modulation (cf. panel c), when walking slowly. The white circles display the onset of an image in the rapid serial visual presentation (RSVP) stream, within the phases of locomotion, and specific onset in trial-time denoted by the vertical arrows at bottom. **(b)** Grand mean histograms for all step durations across participants (N = 19). Slow walking conditions resulted in steps of longer duration. **(c)** To allow pooling of the data and comparison between walking speeds, each stride (two steps) was resampled to a normalised range of 1–100%. **(d)** Target presentation density was approximately uniform over the step cycle. **(e and f)** Heat maps of recorded eye directions (in world coordinates, centred on the direction of travel) averaged across participants for the slow and natural walking conditions. d.v.a. = degrees of visual angle

We first examined the differences in eye-movement behaviour and gait characteristics when walking at either a slow or a natural speed. As expected, the instructions to walk at a natural or slow walking speed resulted in differences in gait parameters and gaze direction. When walking slowly, participants on average lengthened the duration of their steps and exhibited a decrease in the sinusoidal amplitude of changes to the centre of mass over time (Fig. 2). Figure 2A displays an example trial from one participant at each walking speed, exemplifying the elongated step duration during slow walking conditions. Figure 2B displays the grand average histograms of step durations for all participants in each condition (*N* = 19). On average, our peak detection algorithm recorded a total of 429.9 steps (SD = 85.7) when walking slowly (mean duration = 0.72 s, SD = 0.09). When walking at the natural speed a larger number of steps were recorded overall (mean = 636.7, SD = 104.9), at a shorter duration per step (mean duration = 0.61 SD = 0.06).

In order to compare stride-cycle dynamics across walking speeds, we applied a stride-cycle resampling procedure common to gait and posture research (Davidson et al., 2023, 2024; Gard et al., 2004; Hirasaki et al., 1999; MacNeilage & Glasauer, 2017; Moore et al., 2006). This method resamples the duration of all strides (two-sequential steps) between 1 and 100% stride-cycle completion, a normalisation enabling comparison of performance measures relative to the phase of locomotion. Figure 2C displays the grand-average detrended head height per stride cycle at slow and natural walking speeds for our participant*s*. Approximate locations of swing and stance phases as well as heel-strike and toe-off are indicated. These are based on prior studies that have measured foot pressure and head acceleration simultaneously (Mulavara & Bloomberg, 2002) and define the heel-strike and toe-off points as 10% of step-cycle period before and after mid-stance, respectively (MacNeilage & Glasauer, 2017).

### Locomotion entrains saccade onsets

**Fig. 3:**
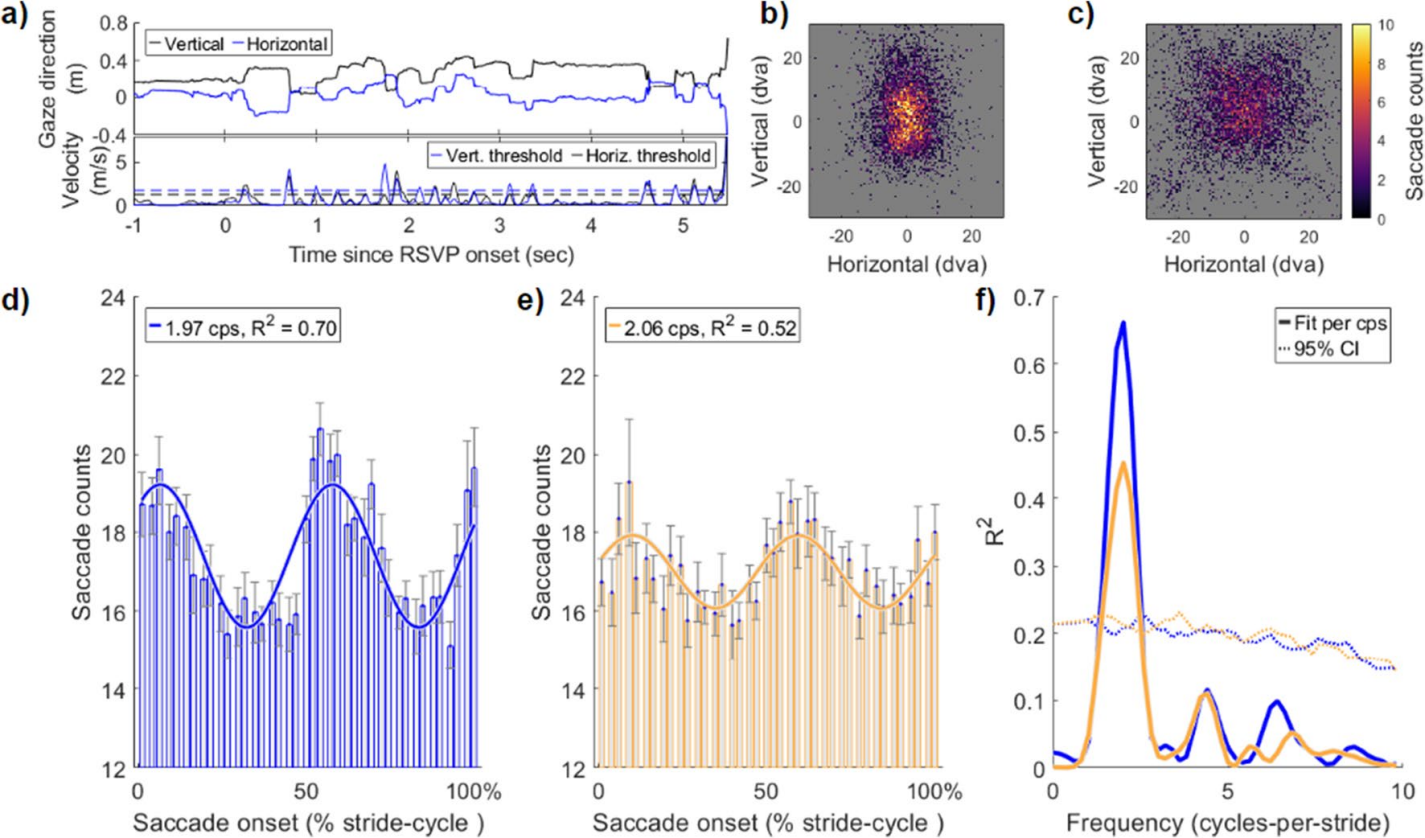
Saccade onsets are entrained to the rhythm of the stride cycle. **(a)** Example gaze direction (black = vertical, blue = horizontal) from one trial. The bottom panel displays the same time-series converted to velocity, and the thresholds for saccade detection on the horizontal and vertical axes (see *Methods*). **(b)** Distribution of all saccade landing positions when walking slowly and **(c)** walking naturally. **(d and e)** Group-level data of saccade-onset timing relative to position in the stride cycle. The best-fitting first-order Fourier model is shown in each figure and approximated two cycles per stride (i.e., the step rate). **(f)** Permutation testing of the Fourier models fitted to saccade reaction time data in (d) and (e). The observed data were fitted with a single-component Fourier model at all frequencies between 0.2 and 10 cycles per stride (in steps of 0.2) and goodness-of-fit (R^2^) was calculated. The dashed line shows the upper bound of the 95% confidence interval calculated by permuting the data

We combined our gait-resampling procedure with a microsaccade detection algorithm to assess the likelihood of saccade onset with respect to the phase of the ongoing stride cycle. Clear oscillations in saccade onset timing are apparent at the group level at both slow and natural walking speeds, with saccade likelihood peaking in the approximate swing phase of each step, immediately after mid-stance. This is clear in Figs. 3D and 3E, which display the group average results (*N* = 19) together with a curve displaying the best-fitting first-order Fourier model (see *Methods*, Eq. 1).

Figure 3F shows the goodness-of-fit (*R*^2^) for all Fourier frequencies in the range 0.2–10 cycles per stride (cps; in steps of 0.2 cps) as well as the upper bound of the 95% CI (dashed line) produced by permuting the data (see *Methods*). Saccade onset during slow walking speeds was best fitted by an oscillation at 1.97 cps (R^2^ = 0.70, range 1.40–2.40 cps above the 95th percentile). Similarly, saccade onset during natural walking speed was best fitted by an oscillation at 2.06 cps (and was significant over the range 1.40–2.40 cps). Together these oscillations in saccade onset indicate that eye movements initiated during walking entrain to the rhythm of footfall, peaking immediately after toe-off and mid-stance during the stride.

Next, we performed participant-level analyses to determine the prevalence of these oscillations in our sample, and to investigate the phase consistency of this entrainment at both walking speeds. the consistency of this saccadic-entrainment phase seen at the group level. For this analysis, we performed the same Fourier fitting and shuffling procedure as at the group level, now per participant, and rejected the null hypothesis when the strength of Fourier fits to observed data exceeded the 95% CI of the participant-level shuffled data. At both walking speeds, significant saccade entrainment at 2 cps was most prevalent (slow speed n = 14/19, normal speed n = 10/19, NHST at 1.5–2.5 cps). A smaller proportion of participants had significant oscillations in saccadic onset at higher cps (slow speed n = 5, natural speed n = 6), resulting in a very high proportion of our total sample displaying saccadic entrainment to step-cycle (slow speed n = 19/19, natural speed n = 16/19).

We formally quantified the population prevalence of these saccadic oscillations using Bayesian inference (Ince et al., 2021). This method returns population prevalence as the MAP estimate, with a 95% highest density posterior interval (HDPI) within which the prevalence estimate resides with 95% probability. The prevalence estimates for saccadic oscillations at 2 cps were high in both the slow-speed (MAP = 0.73, 95% HDPI [0.50, 0.89]) and natural-speed conditions (MAP = 0.51, [0.28, 0.72]).

**Fig. 4:**
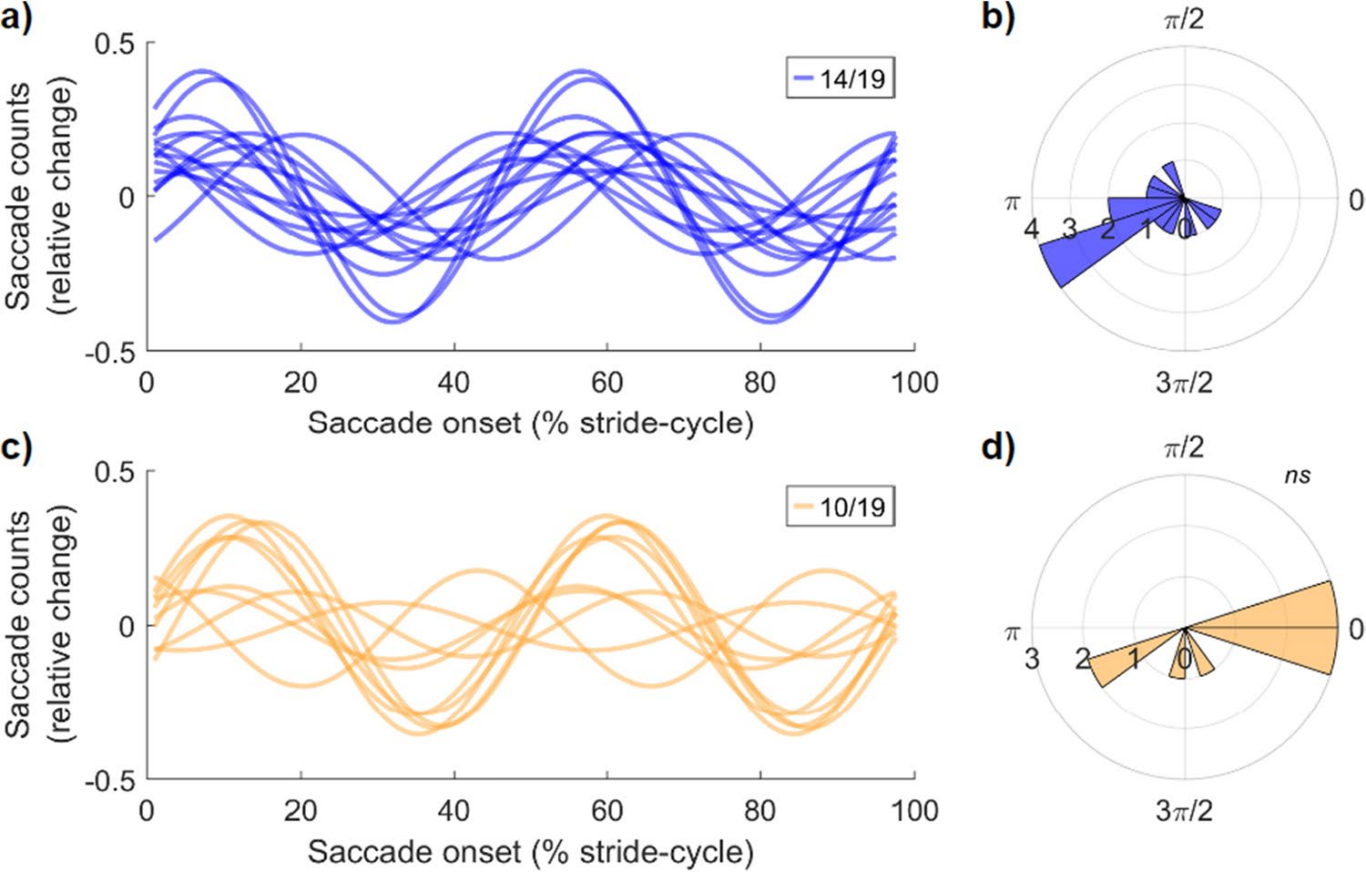
Participant level saccadic oscillations time locked to stride are clustered in phase. **(a)** Fourteen participants had significant oscillations at approximately 2 cycles per stride (cps) when walking slowly. The participant-level Fourier fit is displayed. To compare across participants, the data are expressed in relative change from the participant mean. **(b)** The polar plot of phase angles for the participant oscillations in (a). **(c and d)** Same data for significant oscillations (n = 10) at approximately 2 cps when walking at a natural pace

We next inspected whether the phases of these saccadic oscillations were consistent across participants. For those with significant saccadic oscillations at 2 cps (slow speed n = 14/19, natural n = 10/19), the best-fitting Fourier model was calculated and the phase of each was retained for circular tests of non-uniformity. We observed significant phase clustering at both walking speeds, indicating that saccade onset was most likely around the time of footfall. Rayleigh’s test of non-uniformity confirmed that the phase distribution for saccadic oscillations at slow speeds was non-uniform (Z = 5.49, *p* = 0.003), but not at the natural speed (Z = 2.77, *p* = 0.059). Figure 4 displays a summary of these results.

### Walking speed alters the topography and timing of time–frequency power

**Fig. 5:**
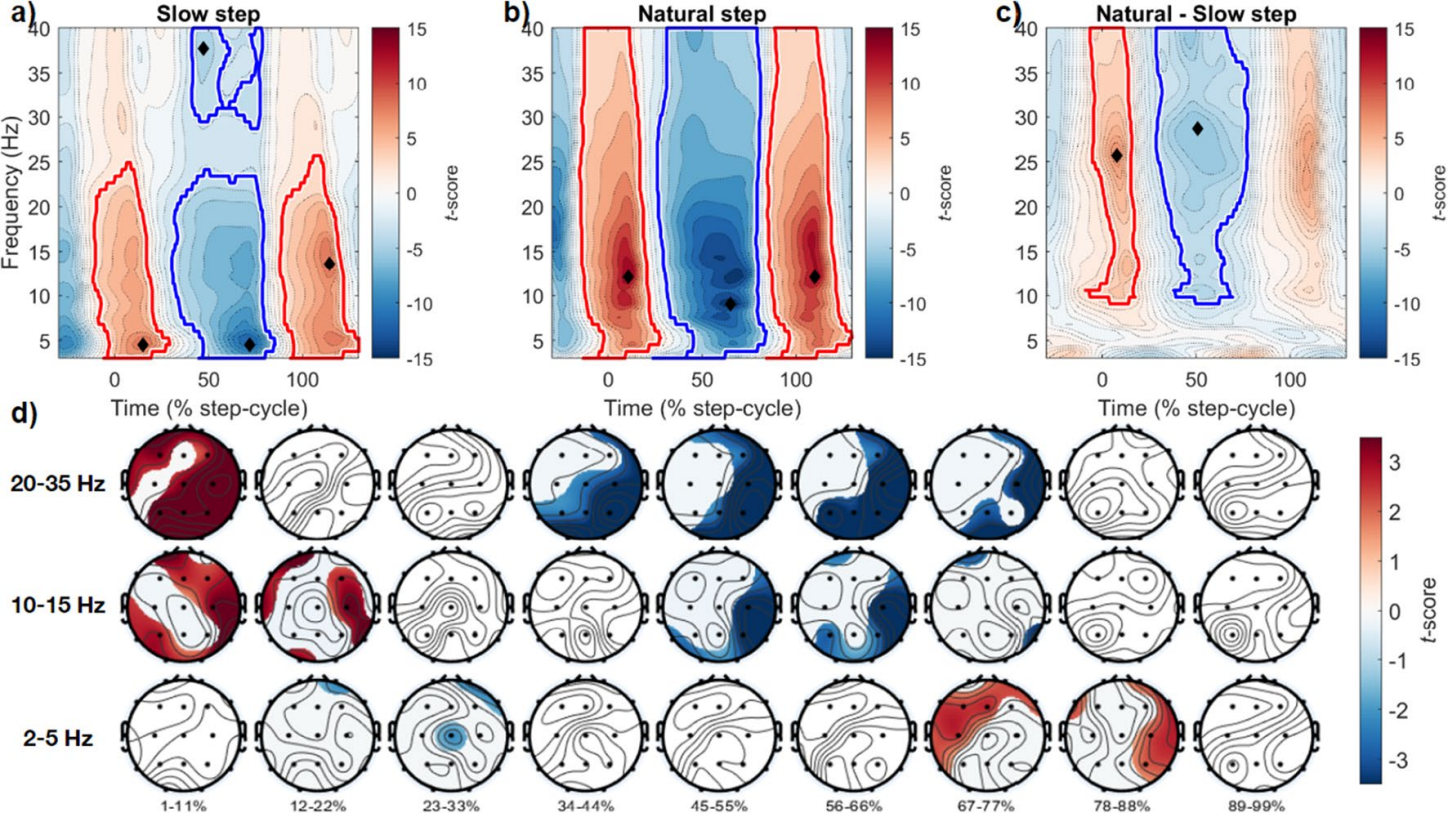
Time–frequency power over the step cycle, comparing slow and natural walking speeds. **(a)** Average time–frequency power from all channels, when walking slowly. Power changes were normalised to percent relative change and significant clusters were compared to the entire epoch baseline and are displayed in each panel (see *Methods*). Red clusters denote increases compared to baseline, blue clusters a decrease compared to baseline. Within each cluster, the time (% step cycle) and frequency (Hz) of the peak effect are displayed with a black diamond. **(b)** Time–frequency power changes during natural walking, other conventions as in (a). **(c)** Comparison of time–frequency activity between walking speeds (natural minus slow). **(d)** Topoplots displaying the scalp distributions of differences between walking speeds averaged within specific frequency bands. The bottom, middle and top rows show power differences at 2–5 Hz, 10–15 Hz and 20–35 Hz, respectively. Each column represents average activity in 9 step cycle percentile bins. Significant differences (p < 0.05, cluster corrected) are unmasked, non-significant regions are overlaid with white masks

Having established that the speed and phase of locomotion alter saccade onset probability, we next analysed the changes in time–frequency power over a broad frequency range at slow and natural walking speeds. We were particularly motivated to extend previous research that focused on alpha-band power at occipital electrodes (Cao et al., 2020) by investigating the topographic changes in time–frequency power over a broad range of frequencies according to the speed and phase of locomotion. Consistent with prior research that confined participants to treadmill walking (Gwin et al., 2011), we observed pronounced fluctuations in power across a range of frequencies during each step cycle (Figs. 5A and 5B; note that the x-axis shows the period of a single step, not a two-step stride cycle). These changes can be summarised as a broadband increase in power around the time of footfall, with a large decrease occurring in the swing phase at each walking speed. Interestingly, although the clusters of activity spanned a broad frequency range, the strongest effects were found at lower frequencies, predominantly in the theta (3–7 Hz) and high-alpha (12–15 Hz) bands.

More specifically, the average from all electrodes revealed that during slow walking a significant positive cluster (*p*_cluster_ < 0.001) increased in power, which spanned 3–24.9 Hz, during the time ranging from -11% to 29% step-cycle completion. This increase was maximal at 4.5 Hz, 15% of the way through the step cycle (*t*(18) = 8.55). Following this, a significant negative cluster (*p*_cluster_ < 0.001; 3–24.1 Hz, 31–86% step cycle) occurred which was maximal at 4.5 Hz at 72% of the step cycle (*t*(18) = -11.43). A simultaneous negative cluster was present at higher frequencies during the swing phase of slow steps (*p*_cluster_ < 0.001; 29–40 Hz, 41–79% step cycle; max at 38 Hz, 47% step cycle; *t*(18) = -4.31). A final significant positive cluster was also present approaching the time of footfall (*p*_cluster_ < 0.001; 3–26.6 Hz, 88–130%), which was maximal at 13.6 Hz (115% step cycle; *t*(18) = 8.74).

Similar results were obtained for the time–frequency dynamics during natural walking, with sequential positive (*p*_cluster_ < 0.001; 3–40 Hz, -15–28% step cycle, max at 12 Hz, 11% step cycle, *t*(18) = 14.18), negative (*p*_cluster_ < 0.001; 3–40 Hz, 23–84% step cycle, max at 9 Hz, 65% step cycle, *t*(18) = -14.68) and positive clusters (*p*_cluster_ < 0.001; 3–40 Hz, 84–130% step cycle, max at 12 Hz, 110% step cycle, *t*(18) = 13.61). Notably, however, the peak activity in these clusters occurred at slightly higher frequencies, in the upper alpha range.

We also calculated the difference in time–frequency power between natural and slow walking conditions. This analysis revealed significant differences in higher frequency ranges, specifically a positive cluster at step onset (*p*_cluster_ < 0.001; 8.2–40 Hz, -13–21% step cycle, max at 25.6 Hz, 8% step cycle, *t*(18) = 7.03), a negative cluster during the swing phase (*p*_cluster_ < 0.001; 8.2–40 Hz, 24–80% step cycle, max at 28.8 Hz, 51% step cycle, *t*(18) = -5.51), and positive cluster in the approach to heel-strike (*p*_cluster_ < 0.001; 9.8–40 Hz, 94–118% step cycle, max at 23.4 Hz, 109% step cycle, *t*(18) = 6.27). Figures 5A–C display a summary of this data.

After observing strong modulations in both low- and high-frequency ranges in the channel-averaged data, we proceeded to investigate the spatial distribution of time–frequency power within specific frequency bands over the step cycle. For this analysis, we focused on the frequency ranges 2–5 Hz, 10–15 Hz and 20–35 Hz, and averaged activity within these ranges over nine-step cycle percentile bins (e.g., 1–11%, 12–22%, …, 89–99% step cycle completion). Figure 5D displays a summary of this data. Most noteworthy are the distinct times and regions for significant activity within each frequency band. For example, lower frequency power (2–5 Hz) is greater during natural walking than slow walking in the approach to foot-fall (67–77%, 78–88% step cycle completion) in predominantly frontocentral and frontotemporal electrode locations. There are no significant differences between walking speeds at this lower frequency range during the swing-phase of each step, in contrast to the higher frequency bands displayed. We return to these characteristics in the *Discussion*.

### Saccade-locked EEG is modulated by step cycle phase and walking speed

**Fig. 6:**
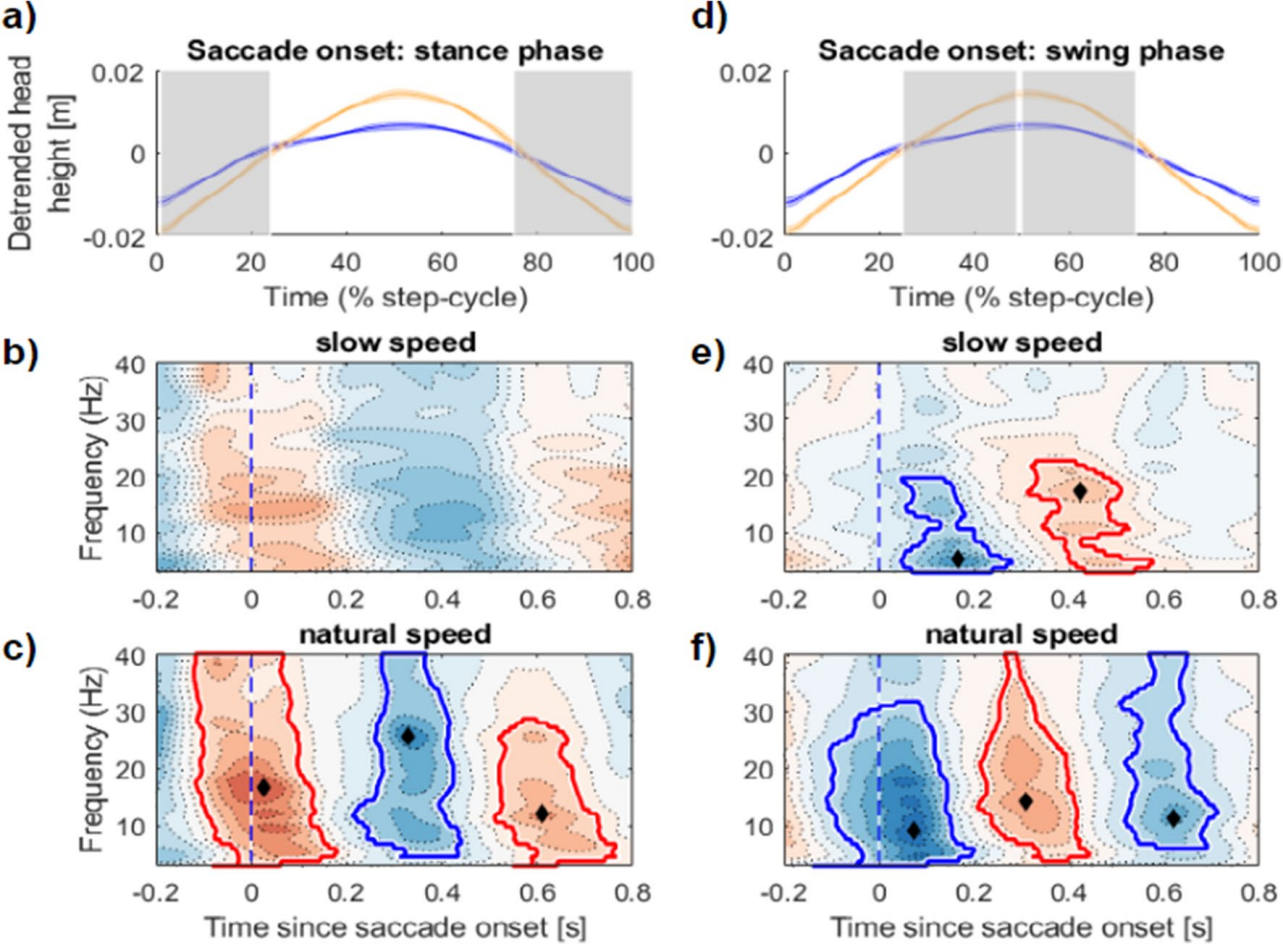
Saccade-locked EEG time–frequency dynamics during locomotion are modulated by step-cycle phase. **(a)** The average detrended change in head height over all participants (blue = slow walk speed, yellow = natural walk speed). The shaded patch denotes the region (in step cycle %) of pooled saccade onsets used in the saccade-locked EEGs in (b) and (c). **(b)** Saccade-locked EEG from the approximate stance phase of the step cycle (1–24%, 75–100%) when walking slowly. **(c)** Saccade-locked EEG in the same approximate stance phase; increases in time–frequency power are shown in red, decreases in blue, with peak frequency and time point of the cluster displayed with a black diamond. **(d–f)** Shows the same data format as in (a)***–***(c) repeated for the approximate swing phase of the step cycle. All clusters corrected for multiple comparisons using non-parametric permutation tests (Maris & Oostenveld, 2007)

Finally, we turned to saccade-locked time–frequency activity, focusing on the changes during the swing phase and approach to footfall of each step. For this analysis, all saccades occurring in either the slow or the natural walking conditions were epoched -200 to 800 ms relative to saccade onset, and the power in each frequency band was normalised to the baseline activity within each frequency across all saccade locked epochs. Similar to the step cycle EEG described above, we observed significant increases and decreases in broadband time–frequency power at both walking speeds, and relative to the phase of step cycle completion. Our analysis here serves to assess the consistency of changes in time–frequency power when re-aligning to saccade onsets in the two main phases of the step-cycle (Fig. 6).

When walking at natural speeds, saccade onsets within the stance phase were accompanied by a significant increase in power at approximately 16.6 Hz (*p*_cluster_ = 0.0005, 3–40 Hz, -120–185 ms, max t(18) = 10.29), followed by a significant decrease in power strongest at 25 Hz (*p*_cluster_ = 0.0035, 4.5–40 Hz, 213–440 ms, max t(18) = -9.15).This sequential increase and decrease in time–frequency power is to be expected given the patterns observed over the step-cycle (cf. Figure 5), although notably, when aligned to saccade onset these effects are now maximal at higher frequencies, and were not significant at slow walking speeds, potentially due to an increase in gait variability when walking slowly.

To further examine this interaction, we repeated this analysis when aligning to saccade onsets in the swing phase of each step. Saccade-locked EEGs aligned to the approximate swing phase of each step were downshifted in frequency. For example, the significant decrease in power occurring in the swing phase when slow walking peaked at 5.2 Hz (*p*_cluster_ < 0.033, 3–19.6 Hz, 46–280 ms, max t(18) = -8.18). Likewise the decrease in power in the approximate swing phase when walking at natural speeds peaked in magnitude at the lower frequency of 9 Hz (*p*_cluster_ < 0.001, 3–31.7 Hz, -140–200 ms, max t(18) = -11.01). In summary, realigning saccade-locked EEGs revealed distinct clusters of activity that interacted with walking speed, and depended on the phase of locomotion. This analysis underscores the importance of accounting for step-cycle phase when interpreting saccade-locked EEGs in moving participants, a consideration we return to in our *Discussion*.

## Discussion

In this study we measured eye movements and EEG activity in observers doing a visual oddball detection task. A critical additional feature was that our observers were active while the measurements were being made, walking back and forth along a path at two walking speeds. Doing the task while engaged in natural active behaviour is important because we are fundamentally active creatures, constantly reaching, head-turning, walking and moving our eyes as we engage in our daily routines and complete relevant tasks. It is therefore important to study how active behaviour might alter cognitive and perceptual performance, and how one type of action might influence another for a holistic understanding to be achieved. With this principle in mind, we designed the current experiment around a walking participant using new advances such as mobile EEG and wireless VR with integrated eye-movement recordings and 3D position tracking. Our principal focus was whether the onset of saccades would entrain to the rhythm of the step-cycle, as previous research has indicated. Specifically, we tested this at two walking speeds and during a task that did not require visually guided stepping. We complement this study with simultaneous recordings of mobile-EEG to examine the relative contributions of locomotor and visuomotor behaviours on neural activity during everyday action.

Our results confirm that important differences do emerge in behaviour and neural activity when measured during action. Specifically, we showed that both the likelihood of making saccades and EEG time–frequency power are modulated rhythmically over the gait cycle, producing an oscillation that matched the step rate (approximately 2 Hz). Moreover, the saccadic and EEG oscillations show a consistent phase relationship with the gait cycle (Figs. 3, 4 and 5), indicating both are linked to gait. By comparing saccade onsets and EEG at two walking speeds, we confirmed that saccadic and EEG oscillations are linked to the phase of the stride-cycle by showing they entrain to the rhythm of footfall even at a slower pace. We also observe separable dynamics to the time–frequency power simultaneously recorded during walking at different speeds, which provide strong considerations for interpreting saccade-locked EEG during locomotion, and mobile EEG in general.

### Saccade onsets entrain to the rhythm of footfall

Participants in our task were required to walk in a straight line while fixating a central cross and monitored eight peripheral target locations during an oddball task. Targets appeared every 200 ms (~ 5 Hz), with onsets that were approximately evenly distributed across the step cycle (Fig. 2D). Despite this uniform presentation rate, we observed strong modulations to the likelihood of saccade onsets that closely matched the step cycle frequency, with a period of approximately 2 cps.

The timing of these phasic changes to the likelihood of saccade onset are noteworthy for several reasons. Earlier work has shown that eye movements precede leg movements with a consistent delay when precision stepping is required (Hollands & Marple-Horvat, 2001), and that this coupling persists in the dark (Cao et al., 2020; Hollands & Marple-Horvat, 2001), suggesting a close functional relationship between these motor commands. In contrast to this tight coupling, Cao et al. (2020) observed that eye movements were not coupled to leg movements during slow walking yet acknowledged this could be due to a paucity of steps in their slow-walking conditions (~ 1.25 min total testing time). In our task, hundreds of steps were recorded at both walking speeds, allowing us to examine the modulation of saccade probability over the phases of the stride-cycle in fine temporal detail. This improvement has revealed that eye movements are entrained during slow walking, and at a stronger modulation than when walking naturally. We additionally have shown that the changes in saccade probability are rhythmic in nature and cluster in phase alignment across participants.

This phase clustering is relevant to prior work investigating phasic demands within the step cycle. Despite the subjectively smooth and continuous experience of walking, the rhythm of walking is supported by ballistic periods of heightened sensory demand. For example, in the swing phase immediately after toe-off, the predictability of head movements are at their lowest (MacNeilage & Glasauer, 2017), leading to increased vestibular demand in order to maintain balance and posture (Bent et al., 2004; Dakin et al., 2013). Similarly, recent work tracking eye movements during movement through challenging terrain has shown an increase in gaze density for locations approximately two steps ahead (Bonnen et al., 2021; Matthis & Fajen, 2014; Matthis et al., 2018), and that information about the terrain must be viewed within a critical window (prior to heel-strike) for smooth locomotion to continue (Matthis & Fajen, 2013; Matthis et al., 2017). These changes in demand coincide with the period of heightened saccade onset probability that we report. Although in our task saccades were not necessary only for smooth locomotion, it is likely that their occurrence helps to orient the body in an uncertain environment to guide locomotion. Indeed, we observed an increased modulation in the strength of saccade onset probability when walking slowly – a task that is known to place additional demands on sensory and cognitive systems (Akaiwa et al., 2022; Al-Yahya et al., 2011; Lajoie et al., 2016). Future research may further increase sensory uncertainty to investigate these modulations in saccade onset probability.

In a related point, it is plausible that visuo-vestibular conflicts may contribute to our results – as our design capitalised on the use of a head-fixed display which contrasts with the typical world-fixed coordinate system of our saccades. While this arrangement is not the norm, it will become increasingly important to understand as new technology is introduced using augmented reality devices and head-mounted displays. By using a head-fixed display, we could ensure that the viewing distance to each image remained constant as participants completed each trial, and investigate whether an interaction between step-cycle and saccade onset exists when saccades are not needed to guide visually guided stepping. Future experiments could investigate how eye movements are coordinated with step behaviour when changing distance to a world-fixed target, and whether the entrainment we have identified also depends on viewing distance.

### Distinct time–frequency power changes that depend on walking speed

We observed significant changes in time–frequency power that were also coupled to the phases of locomotion. Previous examinations of mobile-EEG activity have noted similar large-scale fluctuations in power. In the first study to report intra-stride fluctuations of electrocortical activity during walking, Gwin et al. (2011) identified broadband and sequential increases and decreases in event-related spectral perturbations. Using a high-density EEG array and guided ICA (independent component analysis), these fluctuations were spatially localised to anterior cingulate, parietal and sensorimotor cortex, with no differences between activity at two speeds of treadmill walking (0.8 and 1.25 m/s). We also observed strong fluctuations in alpha- and beta-band activity that were phase-locked to the procession of the step cycle, which unlike this previous research significantly differed at each walking speed. More specifically, the fluctuations in power were maximal in different frequency bands depending on walking speed, peaking in the approximate theta band when walking slowly, and approximate alpha band at natural walking speeds (Fig. 5). We also recorded changes in overall power when comparing between speeds (cf. Figure 5C), yet these effects could partially be driven by the increased variance of step durations when walking slowly, or the asymmetry in step counts. As such, future research could endeavour to equate step counts between conditions, and we here focus instead on the frequency of peak effects within each respective condition.

The frequency of these peak effects in the theta and alpha range are noteworthy for several reasons. As has been noted in the past (Castermans et al., 2014; Gramann et al., 2011, 2014; Gwin et al., 2010), it is critical to remove motion signals and potential artefacts prior to interpreting underlying scalp activity, particularly with respect to activity in the low-delta and high-gamma range, for contamination due to the step rate and muscle activity, respectively (Castermans et al., 2014). Our procedure incorporated highly conservative exclusion criteria and stringent artefact removal and observed strongest effects outside the frequency of the step-rate (> 2 Hz), and below high-gamma fluctuations scrutinised in prior research (< 50–150 Hz). When analysing the topography of this frequency-band activity, we also observed a relative increase in frontal theta-band activity at natural walking speeds, just prior to heel-strike (Fig. 5D). This change in frontal theta is reminiscent of prior reports examining reactive changes to balance and posture, where it has been proposed that theta dynamics may represent a process of action monitoring to maintain balance (Stokkermans et al., 2022a, 2022b).

### Saccades and EEG

Lastly, we viewed changes in the EEG signal around saccades, which were dominated by whether those saccades were in the swing or stance phases of the step cycle, during natural and slow walking. We found that saccade-locked EEG power increased during swing and decreased during approach to heel-strike, more strongly when walking at natural speeds compared to slow. These results underscore the importance of accounting for step-cycle phase and walking speed when interpreting saccade-locked EEG in future experiments. The realignment of saccade-locked EEG to swing and stance phases clearly demonstrated that changes in time–frequency power were driven by the demands of locomotion and interacted with walking speed.

Given the practical challenges of recording eye movements and EEG during free movement, it is unsurprising that there are few comparable studies. Indeed, in the present sample, 15 participants were excluded for incomplete or missing data (e.g., owing to signal drop-out), highlighting the precarious and time-intensive nature of collecting mobile and wireless EEG data. However, static work relating eye movements to EEGs highlights their close relationship. Liu et al. (2023) show changes in ipsilateral alpha power during microsaccades, arguing that even very subtle shifts in gaze can alter signals associated with visual and spatial cognition. This relationship also appears to go in the other direction. Staudigl et al. (2017) used magnetoencephalography (MEG), a close cousin to EEG, to show that posterior alpha phase predicts saccades during memory encoding. That is, saccades may both prime changes in low-frequency power and be triggered by them, in regions that are critical for interpreting our visual world. Here, we show that saccade-related changes in EEG interact with walking speed, and that we can observe this over and above step-cycle-related changes. Together, the co-registration of eye and body movements with EEG demonstrates a way towards understanding motor and perceptual processes in rich neural data during natural movement.

## Conclusion

Overall, our results make an important general point in showing that extrapolating knowledge obtained from traditional experiments to predict outcomes in richer, active environments is not a valid extension. The current knowledge base was very largely built on data from passive observers sitting immobile in darkened laboratories who respond to simple stimuli without the distraction of real-world clutter and the competing demands of being active. This approach has undoubtedly been a rich and successful one but has led to an artificially narrow understanding. On this view, we would not expect that eye movement and EEG activity, as well as visual performance on the oddball task, would show oscillations at the step rate when measured in a walking participant. It is indeed rather surprising to find oscillatory results given that the visual task was an uninterrupted one that required continual monitoring and that walking seems a near-effortless constant activity. Yet, despite this, clear modulations were found, all linked to the gait cycle.

These results have important implications. (1) Because locomotion imposes a rhythmic oscillation on neural, saccadic and perceptual activity, it therefore creates good and bad phases for perceptual performance and saccadic behaviour. Saccades, for example, are more likely to occur at certain moments than others, peaking just after footfall. It is likely this temporal clustering of eye movements would have consequences for tasks such as visual search and change detection, and this could be explored in future experiments. Similarly, the rhythm of walking imposes an oscillation on brain activity that needs to be taken into account when analysing EEG data. (2) A second, more general, implication is to underscore the importance of conducting experiments in active observers in more realistic contexts. The technical hurdles that previously made such experiments very challenging to implement have largely been overcome, and we can now co-register multiple information streams such as EEG and eye movements and various biomarkers in active observers behaving in rich virtual or real environments. Experiments conducted along these lines will add new layers to our knowledge base as more active and realistic features are added to experiments which will, in turn, help bridge the gap between lab-based experiments and real-world applications.

## Data Availability

Raw data and materials are available from the corresponding author upon reasonable request.

## Code availability

Analysis codes are available from the corresponding author upon reasonable request.
